# Poly[dichloridobis[μ-1-(4-pyridylmeth­yl)-1*H*-1,2,4-triazole]copper(II)]

**DOI:** 10.1107/S160053680900645X

**Published:** 2009-02-28

**Authors:** Zhu-Lai Li, Jian Wang, Xiu-Zhi Xu, Xiao Ye

**Affiliations:** a Faculty of Pharmacy Fujian Medical University, Fuzhou, Fujian 350004, People’s Republic of China

## Abstract

The title coordination polymer, [CuCl_2_(C_8_H_8_N_4_)_2_]_*n*_, arose from a layer-separated diffusion synthesis at room temperature. The Cu atom (site symmetry 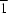) is coordinated by two chloride ions and four N atoms (two from triazole rings and two from pyridyl rings) in a distorted *trans*-CuCl_2_N_4_ octa­hedral arrangement. The bridging 1-(4-pyridylmeth­yl)-1*H*-1,2,4-triazole ligands [dihedral angle between the triazole and pyridine rings = 68.08 (8)°] result in a two-dimensional 4^4^ sheet structure in the crystal.

## Related literature

For background on the synthesis and structures of coordination polymers, see: Carlucci *et al.* (2000 ▶, 2004 ▶); Effendy *et al.* (2003 ▶); Evans *et al.* (1999 ▶); Huang *et al.* (2006 ▶); Liu *et al.* (2005 ▶); Moulton & Zaworotko (2001 ▶); Ranford *et al.* (1999 ▶); Sharma & Rogers (1999 ▶).
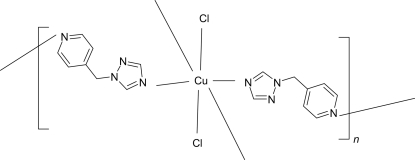


## Experimental

### 

#### Crystal data

[CuCl_2_(C_8_H_8_N_4_)_2_]
*M* *_r_* = 454.81
Monoclinic, 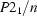

*a* = 7.5112 (5) Å
*b* = 16.0876 (9) Å
*c* = 8.3390 (6) Å
β = 116.469 (2)°
*V* = 902.03 (10) Å^3^
*Z* = 2
Mo *K*α radiation
μ = 1.53 mm^−1^
*T* = 293 K
0.30 × 0.20 × 0.15 mm

#### Data collection

Siemens SMART diffractometer
Absorption correction: multi-scan (*SADABS*; Siemens, 1996 ▶) *T*_min_ = 0.88, *T*_max_ = 1.00 (expected range = 0.700–0.795)
6345 measured reflections
2067 independent reflections
1864 reflections with *I* > 2σ(*I*)
*R*_int_ = 0.017

#### Refinement

*R*[*F* ^2^ > 2σ(*F* ^2^)] = 0.036
*wR*(*F* ^2^) = 0.087
*S* = 1.01
2067 reflections
124 parameters
H-atom parameters constrained
Δρ_max_ = 0.81 e Å^−3^
Δρ_min_ = −0.56 e Å^−3^

### 

Data collection: *SMART* (Siemens, 1996 ▶); cell refinement: *SAINT* (Siemens, 1996 ▶); data reduction: *SAINT*; program(s) used to solve structure: *SHELXS97* (Sheldrick, 2008 ▶); program(s) used to refine structure: *SHELXL97* (Sheldrick, 2008 ▶); molecular graphics: *SHELXTL* (Sheldrick, 2008 ▶); software used to prepare material for publication: *SHELXL97*.

## Supplementary Material

Crystal structure: contains datablocks I, global. DOI: 10.1107/S160053680900645X/hb2898sup1.cif

Structure factors: contains datablocks I. DOI: 10.1107/S160053680900645X/hb2898Isup2.hkl

Additional supplementary materials:  crystallographic information; 3D view; checkCIF report

## Figures and Tables

**Table 1:** Selected bond lengths (Å)

Cu1—N3	2.034 (2)
Cu1—N4^i^	2.087 (2)
Cu1—Cl1	2.7167 (7)

Symmetry code: (i) 
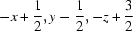
.

**Fig. 1.**
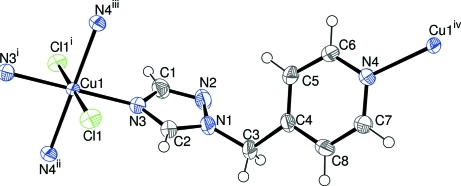
A view of the asymmetric unit of (I), showing 50% probability displacement ellipsoids, expanded to show the Cu geometry. Symmetry codes: (i) 1–x, –y, 1–z; (ii) 1/2–x, y–1/2, 3/2–z; (iii) x+1/2, 1/2–y, z–1/2; (iv) 1/2–x, 1/2+y, 3/2–z.

**Fig. 2.**
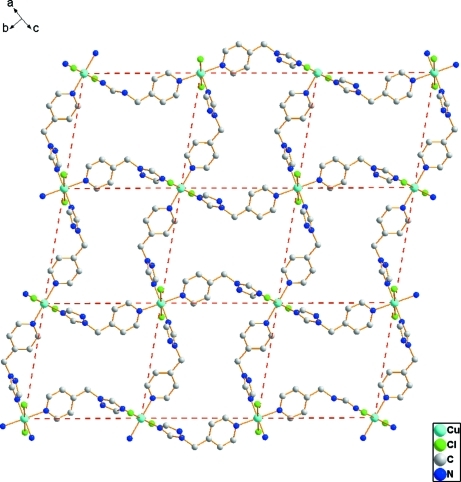
The two-dimensional extended structure of (I), constructed of rhombus-shaped grids.

## supplementary crystallographic information

### Comment

In the research of supramolecular chemistry, a great interest has recently been focused on the crystal engineering of coordination frameworks due to their intriguing architectures, new topologies, intertwining phenomena and potential applications in microelectronics, nonlinear optics, ion exchange, molecular selection, molecular separation and recognition (Carlucci *et al.*, 2000; Evans *et al.*, 1999; Ranford *et al.*, 1999; Sharma *et al.*, 1999). The structural motifs of coordination polymers rest on several factors, but the choice of appropriate ligands is no doubt the key factor because it has an obvious influence on the topologies of coordination polymers and behavior of the molecules. Some flexible ligands, such as bis(triazole), bis(benzotriazole) and bis(pyridyl) alkyl, have been utilized to construct coordination polymers with aesthetics and useful properties (Moulton *et al.*, 2001; Carlucci *et al.*, 2004; Effendy *et al.*, 2003), but the symmetry greatly limits the novelty and variety of the configuration.

Recently, our group have focused on the design and synthesis of some flexible unsymmetric ligands(Liu *et al.*, 2005; Huang *et al.*, 2006), and we have got a new heterocyclic ligand pyta [pyta = *N*-(4-pyridylmethyl)(1,2,4-triazole)]. In order to explore the architectural styles and other chemistry of this kind of ligands, we selected copper chloride as representative subject for stereoregular coordination. Among our attempts, a new polymer, namely [Cu(pyta)_2_Cl_2_]n,(I), was obtained as crystals suitable for single-crystal X-ray analysis.

The crystal structure of (I) is illustrated in Fig.1. The asymmetric unit contains one copper atom lying on an inversion centre, one chloride ion donor and one pyta bridging group. The Cu(II) center lies in an octahedral [CuN_4_Cl_2_] environment with the axial positions occupied by two chloride ions and the equatorial positions occupied by two *trans* triazolium nitrogen atoms and two *trans* pyridyl nitrogen atoms, each of which respectively belongs to four different pyta ligands. The bond angles about the Cu(1) octahedron range from 87.20 (8)° to 92.80 (8)° and deviate slightly from those of a perfect octahedron. The Cu—N bond lengths are in the range 2.034 (2) - 2.087 (2) Å. Due to the existence of the CH_2_ spacer between the triazole and the pyridyl ring, sufficient flexibility make it possible for pyta to be twisted to meet the requirment of coordination geometries of Cu(II) center with the N(1)—C(3)—C(4) torsion angle 115.6 (2)° and the dihedral angle 68.08 (8)°.

The polymer results in an infinite two-dimensional rhombohedral sheet containing 36-membered sandglass rings, as shown in Fig.2. The *sp*-3 configuration of C(3) forces the pyta ligand to be non-linear, generating the nonlinear grid sides and thereby the sandglass grids. Every complementary four [Cu_4_(pyta)_4_] grids are joined together by sharing the copper apices to give the 4^4^ two-dimensional structure with a side length of 10.495 Å and a diagonal measurement of about 13.483 × 16.088 Å.

### Experimental

A solution of pyta (0.016 g, 0.10 mmol) in MeOH (5 ml) was carefully layered on a solution of CuCl_2_.2H_2_O (0.017 g, 0.10 mmol) in H_2_O (5 ml). Diffusion between the two phases over about twenty days produced blue prisms of (I) (yield 0.013 g, 28.6%). Anal. Calcd for C_16_H_16_N_8_Cl_2_Cu (%): C, 32.16; H, 2.74; N, 19.02. Found: C, 32.78; H, 2.45; N, 19.30. IR (KBr, cm^-1^): 3700–3500 (*s*), 2374 (*m*), 1488 (*m*), 1425 (*s*), 1409 (*s*), 1273 (*m*), 1185 (*m*), 1169 (*m*), 1025 (*s*), 1011 (*m*), 783 (*m*), 659 (w), 452 (w).

### Refinement

The hydrogen atom positions were generated geometrically and refined as riding with U_iso_(H) = 1.2U_eq_(C).

### Crystal data

**Table:**

[CuCl_2_(C_8_H_8_N_4_)_2_]	*F*(000) = 462
*M**_r_* = 454.81	*D*_x_ = 1.674 Mg m^−^^3^
Monoclinic, *P*2_1_/*n*	Mo *K*α radiation, λ = 0.71073 Å
Hall symbol: -P 2yn	Cell parameters from 2134 reflections
*a* = 7.5112 (5) Å	θ = 2.7–27.5°
*b* = 16.0876 (9) Å	µ = 1.53 mm^−^^1^
*c* = 8.3390 (6) Å	*T* = 293 K
β = 116.469 (2)°	Prism, blue
*V* = 902.03 (10) Å^3^	0.30 × 0.20 × 0.15 mm
*Z* = 2	

### Data collection

**Table:**

Siemens SMART diffractometer	2067 independent reflections
Radiation source: fine-focus sealed tube	1864 reflections with *I* > 2σ(*I*)
graphite	*R*_int_ = 0.017
ω scans	θ_max_ = 27.5°, θ_min_ = 3.0°
Absorption correction: multi-scan (*SADABS*; Siemens, 1996)	*h* = −9→6
*T*_min_ = 0.88, *T*_max_ = 1.00	*k* = −20→16
6345 measured reflections	*l* = −10→10

### Refinement

**Table:**

Refinement on *F*^2^	Primary atom site location: structure-invariant direct methods
Least-squares matrix: full	Secondary atom site location: difference Fourier map
*R*[*F*^2^ > 2σ(*F*^2^)] = 0.036	Hydrogen site location: inferred from neighbouring sites
*wR*(*F*^2^) = 0.087	H-atom parameters constrained
*S* = 1.01	*w* = 1/[σ^2^(*F*_o_^2^) + (0.0347*P*)^2^ + 1.5333*P*] where *P* = (*F*_o_^2^ + 2*F*_c_^2^)/3
2067 reflections	(Δ/σ)_max_ < 0.001
124 parameters	Δρ_max_ = 0.81 e Å^−^^3^
0 restraints	Δρ_min_ = −0.56 e Å^−^^3^

### Special details

**Table:**

Geometry. All e.s.d.'s (except the e.s.d. in the dihedral angle between two l.s. planes) are estimated using the full covariance matrix. The cell e.s.d.'s are taken into account individually in the estimation of e.s.d.'s in distances, angles and torsion angles; correlations between e.s.d.'s in cell parameters are only used when they are defined by crystal symmetry. An approximate (isotropic) treatment of cell e.s.d.'s is used for estimating e.s.d.'s involving l.s. planes.
Refinement. Refinement of *F*^2^ against ALL reflections. The weighted *R*-factor *wR* and goodness of fit *S* are based on *F*^2^, conventional *R*-factors *R* are based on *F*, with *F* set to zero for negative *F*^2^. The threshold expression of *F*^2^ > σ(*F*^2^) is used only for calculating *R*-factors(gt) *etc*. and is not relevant to the choice of reflections for refinement. *R*-factors based on *F*^2^ are statistically about twice as large as those based on *F*, and *R*- factors based on ALL data will be even larger.

### Fractional atomic coordinates and isotropic or equivalent isotropic displacement parameters (Å^2^)

**Table:**

	*x*	*y*	*z*	*U*_iso_*/*U*_eq_	
Cu1	0.5000	0.0000	0.5000	0.02592 (13)	
Cl1	0.10912 (10)	0.03685 (4)	0.30125 (10)	0.03818 (18)	
C1	0.6294 (4)	0.07131 (16)	0.8758 (4)	0.0365 (6)	
H1A	0.7634	0.0666	0.9032	0.044*	
C2	0.3175 (4)	0.06044 (15)	0.7390 (4)	0.0307 (5)	
H2A	0.1881	0.0482	0.6555	0.037*	
C3	0.2381 (5)	0.12190 (16)	0.9732 (4)	0.0355 (6)	
H3A	0.3014	0.1057	1.0984	0.043*	
H3B	0.1159	0.0902	0.9147	0.043*	
C4	0.1852 (4)	0.21295 (15)	0.9625 (3)	0.0291 (5)	
C5	0.2897 (4)	0.27713 (16)	0.9343 (4)	0.0358 (6)	
H5A	0.3987	0.2659	0.9130	0.043*	
C6	0.2312 (4)	0.35851 (16)	0.9379 (4)	0.0337 (6)	
H6A	0.3050	0.4011	0.9211	0.040*	
C7	−0.0305 (4)	0.31581 (17)	0.9856 (4)	0.0403 (7)	
H7A	−0.1439	0.3284	0.9991	0.048*	
C8	0.0212 (4)	0.23381 (17)	0.9888 (5)	0.0419 (7)	
H8A	−0.0534	0.1925	1.0084	0.050*	
N1	0.3680 (4)	0.09900 (13)	0.8939 (3)	0.0322 (5)	
N2	0.5650 (4)	0.10725 (15)	0.9833 (3)	0.0378 (5)	
N3	0.4802 (3)	0.04199 (13)	0.7215 (3)	0.0300 (5)	
N4	0.0740 (3)	0.37867 (12)	0.9642 (3)	0.0279 (4)	

### Atomic displacement parameters (Å^2^)

**Table:**

	*U*^11^	*U*^22^	*U*^33^	*U*^12^	*U*^13^	*U*^23^
Cu1	0.0367 (2)	0.0165 (2)	0.0367 (2)	−0.00556 (16)	0.0272 (2)	−0.00450 (16)
Cl1	0.0402 (4)	0.0381 (4)	0.0442 (4)	−0.0050 (3)	0.0260 (3)	−0.0017 (3)
C1	0.0337 (14)	0.0291 (13)	0.0474 (16)	0.0011 (11)	0.0189 (12)	−0.0053 (11)
C2	0.0349 (13)	0.0266 (12)	0.0366 (13)	−0.0025 (10)	0.0214 (11)	−0.0046 (10)
C3	0.0505 (16)	0.0232 (12)	0.0460 (16)	0.0040 (11)	0.0334 (14)	−0.0026 (11)
C4	0.0387 (14)	0.0217 (11)	0.0313 (13)	0.0030 (10)	0.0196 (11)	−0.0027 (9)
C5	0.0411 (15)	0.0286 (12)	0.0522 (17)	0.0053 (11)	0.0337 (14)	0.0005 (11)
C6	0.0393 (14)	0.0264 (12)	0.0478 (16)	0.0000 (11)	0.0305 (13)	0.0015 (11)
C7	0.0376 (15)	0.0259 (13)	0.071 (2)	0.0007 (11)	0.0364 (15)	−0.0021 (13)
C8	0.0444 (16)	0.0223 (12)	0.073 (2)	−0.0047 (11)	0.0383 (16)	−0.0034 (12)
N1	0.0439 (13)	0.0221 (10)	0.0374 (12)	0.0032 (9)	0.0243 (11)	−0.0023 (8)
N2	0.0409 (13)	0.0329 (12)	0.0409 (13)	−0.0017 (10)	0.0195 (11)	−0.0107 (10)
N3	0.0321 (11)	0.0252 (10)	0.0396 (12)	−0.0018 (8)	0.0223 (10)	−0.0039 (9)
N4	0.0330 (11)	0.0195 (9)	0.0375 (11)	0.0024 (8)	0.0212 (10)	0.0018 (8)

### Geometric parameters (Å, °)

**Table:**

Cu1—N3	2.034 (2)	C3—H3A	0.9700
Cu1—N3^i^	2.034 (2)	C3—H3B	0.9700
Cu1—N4^ii^	2.087 (2)	C4—C5	1.379 (4)
Cu1—N4^iii^	2.087 (2)	C4—C8	1.386 (4)
Cu1—Cl1	2.7167 (7)	C5—C6	1.385 (4)
Cu1—Cl1^i^	2.7167 (7)	C5—H5A	0.9300
C1—N2	1.327 (4)	C6—N4	1.334 (3)
C1—N3	1.359 (4)	C6—H6A	0.9300
C1—H1A	0.9300	C7—N4	1.340 (3)
C2—N1	1.327 (3)	C7—C8	1.372 (4)
C2—N3	1.327 (3)	C7—H7A	0.9300
C2—H2A	0.9300	C8—H8A	0.9300
C3—N1	1.450 (3)	N1—N2	1.334 (3)
C3—C4	1.510 (3)	N4—Cu1^iv^	2.0870 (19)
			
N3—Cu1—N3^i^	180.0	H3A—C3—H3B	107.4
N3—Cu1—N4^ii^	92.80 (8)	C5—C4—C8	117.3 (2)
N3^i^—Cu1—N4^ii^	87.20 (8)	C5—C4—C3	125.6 (2)
N3—Cu1—N4^iii^	87.20 (8)	C8—C4—C3	117.0 (2)
N3^i^—Cu1—N4^iii^	92.80 (8)	C4—C5—C6	119.6 (2)
N4^ii^—Cu1—N4^iii^	180.0	C4—C5—H5A	120.2
N3—Cu1—Cl1	89.14 (6)	C6—C5—H5A	120.2
N3^i^—Cu1—Cl1	90.86 (6)	N4—C6—C5	123.1 (2)
N4^ii^—Cu1—Cl1	90.41 (6)	N4—C6—H6A	118.5
N4^iii^—Cu1—Cl1	89.59 (6)	C5—C6—H6A	118.5
N3—Cu1—Cl1^i^	90.86 (6)	N4—C7—C8	123.4 (2)
N3^i^—Cu1—Cl1^i^	89.14 (6)	N4—C7—H7A	118.3
N4^ii^—Cu1—Cl1^i^	89.59 (6)	C8—C7—H7A	118.3
N4^iii^—Cu1—Cl1^i^	90.41 (6)	C7—C8—C4	119.6 (2)
Cl1—Cu1—Cl1^i^	180.0	C7—C8—H8A	120.2
N2—C1—N3	113.3 (2)	C4—C8—H8A	120.2
N2—C1—H1A	123.4	C2—N1—N2	110.8 (2)
N3—C1—H1A	123.4	C2—N1—C3	127.2 (2)
N1—C2—N3	109.5 (2)	N2—N1—C3	121.6 (2)
N1—C2—H2A	125.3	C1—N2—N1	103.1 (2)
N3—C2—H2A	125.3	C2—N3—C1	103.3 (2)
N1—C3—C4	115.6 (2)	C2—N3—Cu1	128.15 (19)
N1—C3—H3A	108.4	C1—N3—Cu1	127.69 (18)
C4—C3—H3A	108.4	C6—N4—C7	116.9 (2)
N1—C3—H3B	108.4	C6—N4—Cu1^iv^	124.24 (17)
C4—C3—H3B	108.4	C7—N4—Cu1^iv^	118.55 (17)

Symmetry codes: (i) −*x*+1, −*y*, −*z*+1; (ii) −*x*+1/2, *y*−1/2, −*z*+3/2; (iii) *x*+1/2, −*y*+1/2, *z*−1/2; (iv) −*x*+1/2, *y*+1/2, −*z*+3/2.

## References

[bb1] Carlucci, L., Ciani, G., Moret, M., Proserpio, D. M. & Rizzato, S. (2000). *Angew. Chem. Int. Ed.* **39**, 1506–1510. 10.1002/(sici)1521-3773(20000417)39:8<1506::aid-anie1506>3.0.co;2-u 10777657

[bb2] Carlucci, L., Ciani, G. & Proserpio, D. M. (2004). *Chem. Commun.* **4**, 380–386. 10.1039/b314322h 14765218

[bb3] Effendy, Marchetti, F. & Pettinari, C. (2003). *Inorg. Chem.* **42**, 112–117. 10.1021/ic025981q 12513084

[bb4] Evans, O. R., Xiong, R. G., Wang, Z. Y., Wong, G. K. & Lin, W. B. (1999). *Angew. Chem. Int. Ed.* **38**, 536–538. 10.1002/(SICI)1521-3773(19990215)38:4<536::AID-ANIE536>3.0.CO;2-3 29711763

[bb5] Huang, M., Liu, P., Chen, Y., Wang, J. & Liu, Z. (2006). *J. Mol. Struct.* **788**, 211–217.

[bb6] Liu, Z., Liu, P., Chen, Y., Wang, J. & Huang, M. H. (2005). *Inorg. Chem. Commun.* **8**, 212–215.

[bb7] Moulton, B. & Zaworotko, M. J. (2001). *Chem. Rev.* **101**, 1629–1658. 10.1021/cr9900432 11709994

[bb8] Ranford, J. D., Vittal, J. J. & Wu, D. (1999). *Angew. Chem. Int. Ed.* **38**, 3498–3501. 10.1002/(sici)1521-3773(19991203)38:23<3498::aid-anie3498>3.3.co;2-6 10602220

[bb9] Sharma, C. V. K. & Rogers, R. D. (1999). *Chem. Commun.* **1**, 83–84.

[bb10] Sheldrick, G. M. (2008). *Acta Cryst.* A**64**, 112–122. 10.1107/S0108767307043930 18156677

[bb11] Siemens (1996). *SMART*, *SAINT* and *SADABS* Siemens Analytical X-ray Instruments Inc., Madison, Wisconsin, USA.

